# Trends and issues in clinical research on satisfaction and quality of life after mastectomy and breast reconstruction: a 5-year scoping review

**DOI:** 10.1007/s10147-023-02347-5

**Published:** 2023-05-09

**Authors:** Miho Saiga, Ryoko Nakagiri, Yuko Mukai, Hiroshi Matsumoto, Yoshihiro Kimata

**Affiliations:** 1 Department of Plastic Surgery, Okayama University Hospital, 2-5-1, Shikata-cho, Kita-ku, Okayama City Okayama, 700-8558 Japan; 2 Department of Plastic Surgery, Okayama Rosai Hospital, Okayama, Japan; 3 Department of Plastic and Reconstructive Surgery, Graduate School of Medicine, Dentistry and Pharmaceutical Sciences, Okayama, Japan

**Keywords:** Patient-reported outcomes, Breast reconstruction, Breast cancer, Quality of life, Satisfaction

## Abstract

Breast reconstruction (BR) aims to improve the satisfaction and quality of life (QOL) of breast cancer survivors. Clinical studies using patient-reported outcomes (PROs) can therefore provide relevant information to the patients and support decision-making. This scoping review was conducted to analyze recent trends in world regions, methods used, and factors investigated. The literature search was conducted in August 2022. Databases of PubMed, MEDLINE, and CINAHL were searched for relevant English-language studies published from 2017 to 2022. Studies involving women with breast cancer who underwent BR after mastectomy and investigated PROs after BR using BR-specific scales were included. Data on the country, publication year, study design, PRO measures (PROMs) used, time points of surveys, and research themes were collected. In total, 147 articles met the inclusion criteria. BREAST-Q was the most widely used, contributing to the increase in the number and diversification of studies in this area. Such research has been conducted mainly in North America and Europe and is still developing in Asia and other regions. The research themes involved a wide range of clinical and patient factors in addition to surgery, which could be influenced by research methods, time since surgery, and even cultural differences. Recent BR-specific PROMs have led to a worldwide development of research on factors that affect satisfaction and QOL after BR. PRO after BR may be influenced by local cultural and social features, and it would be necessary to accumulate data in each region to draw clinically useful conclusion.

## Introduction

Currently, surgical strategies for breast cancer are becoming increasingly diverse, including the development of breast reconstruction (BR) techniques and the widespread of prophylactic mastectomy. BR aims to improve the body image and quality of life (QOL) of breast cancer survivors; however, patients must choose the best treatment for themselves, considering implant-specific issues, donor-site sacrifice, psychological burden, costs, and physical and social rehabilitation. Thus, to help patients make a choice suitable for their situation and preferences, information on the advantages and disadvantages of each option in terms of QOL, complications, and aesthetic outcomes is necessary.

Outcome evaluation using patient-reported outcomes (PROs) is helpful for these areas. It enables scientifically quantify multidimensional outcomes that are only known to the patient, contributes to the consideration of patient-centered treatment strategies, supports decision making, and improves the quality of healthcare [1]. Only objective esthetic and symmetry evaluation has been used to assess BR outcomes in the past; however, with the advent of well-validated BR-specific PRO measures (PROMs) [2, 3], deep understanding of various aspects of patient’s life, such as body image, pain, ease of bra wear, and psychological aspects, has become possible.

Although these BR-specific measures have been incorporated into clinical studies and have deepened research on QOL after BR, the influencing factors are diverse and complex, and the evidence remains insufficient [4–6]. A scoping review of articles published in the last 5 years on this area was conducted to map and organize which world regions, which methods, and which factors were investigated. This review aimed to analyze the trend of studies, rather than study outcomes, to guide future research planning.

## Methods

This scoping review was conducted according to the principles of the Preferred Reporting Items for Systematic Reviews and Meta-Analyses statement [7]. The following question guided the mapping of this scoping review: What world regions, what research methods are being used, and what research questions are being investigated in recent clinical studies on satisfaction and QOL after mastectomy and BR?

### Literature search

The literature search was conducted in August 2022. PubMed, MEDLINE, and CINAHL databases were searched for relevant English-language studies published from 2017 to 2022. The combination of search terms “breast reconstruction” and “breast cancer” with “patient-reported outcomes” or “satisfaction” or “quality of life” was used.

### Selection of eligible studies

The inclusion and exclusion criteria were predetermined to select the relevant studies. The studies were included if it fulfilled all of following criteria: (1) studies involving women with breast cancer or with hereditary breast and ovarian cancer syndrome (HBOC) and underwent therapeutic or prophylactic mastectomy (PM), (2) studies involving women who underwent immediate BR (IBR) or delayed BR (DBR), (3) studies that assessed postoperative satisfaction and QOL using the BR-specific PROMs (namely, BREAST-Q [8, 9], Breast Reconstruction Satisfaction Questionnaire (BRECON-31) [10–12], and the European Organization for Research and Treatment of Cancer Quality of life Questionnaire BRR26 (EORTC QLQ-BRR26) [13, 14], which were assessed as well-developed in a previous study), and examined factors that affect them [2], and (4) any type of BR including autologous and implant-based BR, and secondary procedures such as fat grafting, nipple reconstruction, contralateral symmetrization.

The exclusion criteria were as follows: (1) studies about BR following breast conserving surgery (BCS), (2) studies that compared BR with MT and BCS, and (3) opinion, review, letter, meta-analysis, case report, case series, pre-post study, qualitative study.

Two reviewers (MS and RN) independently screened the title and abstracts to determine whether the studies met the criteria. Disagreements were resolved by further discussion between the two reviewers.

### Data collection, analysis, and reporting of results

After determining studies eligible for inclusion in the final review, data were extracted by a reviewer (MS) to identify the following aspects: (a) region and country of the first author, (b) publication year, (c) study design, (d) PROM used, (e) time point of the PRO survey, and (f) research theme. Endnote and Excel were used for the management and analysis of studies.

The number of publications was described by country, year, and methodology of PRO investigation. The main themes of the study were divided into the following four categories: (i) factors related to reconstructive surgery, (ii) clinical factors related to indication and treatment, (iii) patient factors, and (iv) factors affecting QOL and satisfaction. After the categorization was reconfirmed by another reviewer (RN), the distribution of study themes by region was analyzed.

## Results

**Fig. 1:**
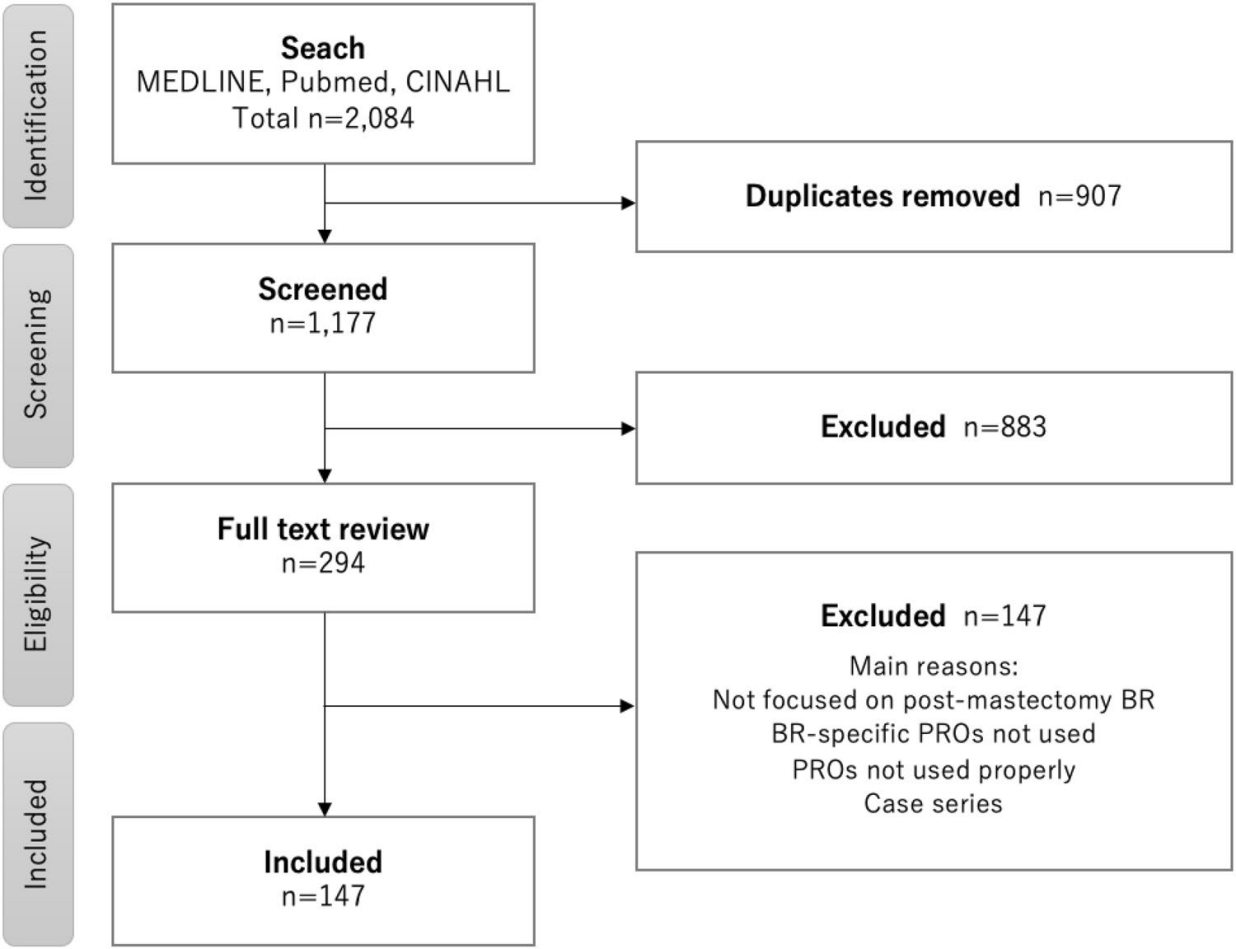
PRISMA flow diagram of the search results

In total, 1177 studies were retrieved from the literature search, 294 full texts were reviewed, and 147 articles were included in the final analysis (Fig. 1).

### Region and publication year

Table 1 shows the countries of affiliation of the first author. Of the 147 studies, 69 (46.9%) were from North America, followed by 57 (38.8%) from Europe. Moreover, 17 (11.6%) papers were from Asia, of which more than half were from China. Very few papers were published from the rest of the world.

**Table 1:** Geographical classification of the included articles

Region, country	No. of articles
**A. North America**	**69 (46.9%)**
USA	67
Canada	2
**B. Europe**	**57 (38.8%)**
UK	14
Italy	14
Netherlands	8
Sweden	7
Spain	5
Germany	4
Belgium	2
Czech Republic	2
Norway	1
**C. Asia**	**17 (11.6%)**
China	9
Taiwan	4
Japan	2
India	1
Saudi Arabia	1
**D. Oceania**	**3 (2.0%)**
Australia	3
**E. South America**	**1 (0.7%)**
Brazil	1

**Fig. 2:**
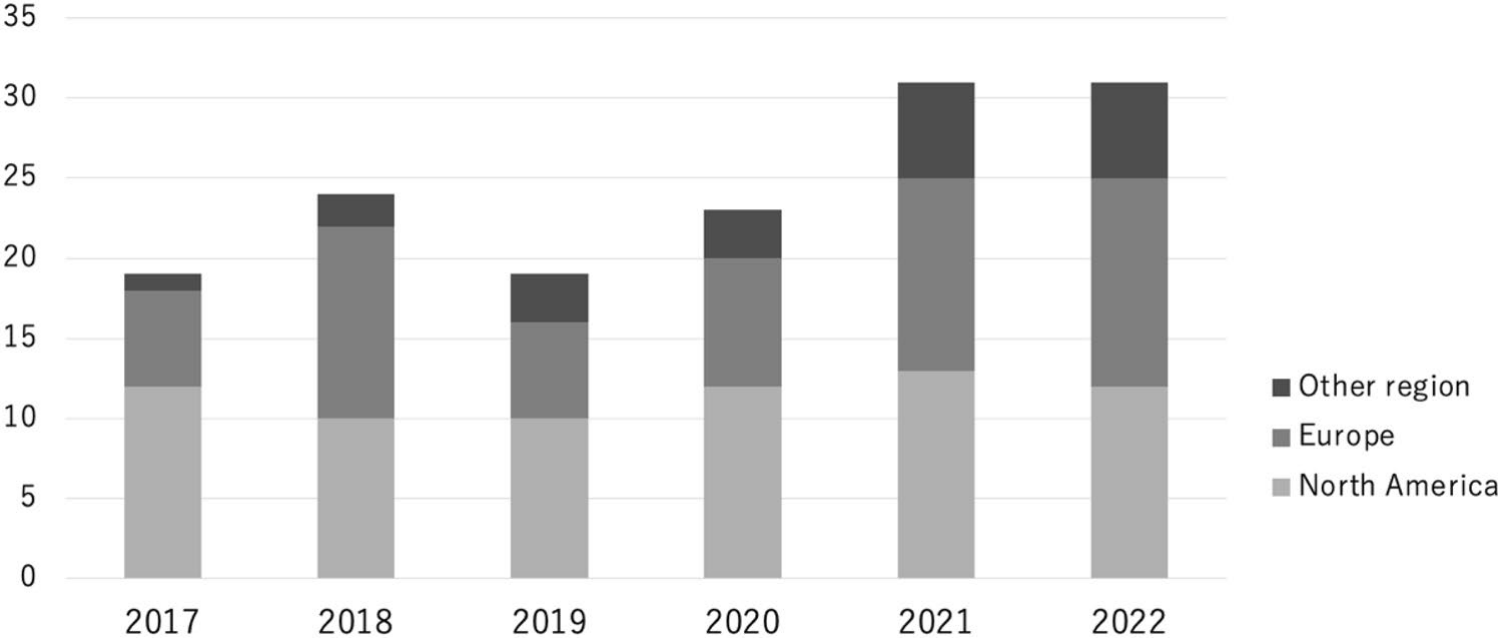
Distribution of studies by world regions from 2017 to 2022. The search was completed in August 2022

As shown in the number of publications each year by region, the number consistently exceeds 10 in North America and is increasing in Europe and other regions (Fig. 2).

### Method to investigate PROs

Table 2 shows the characteristic regarding the methodology of the studies included, which were as follows: 76 (51.7%) cross-sectional studies, 23 (15.6%) retrospective cohort, 42 (28.6%) prospective studies, and 6 (4.1%) randomized controlled trials (RCT). Propensity-score matching analysis was used in three cross-sectional and two retrospective cohort studies.

**Table 2:** Classification by methodology to investigate PROs (study characteristics)

Category	N	(%)
Study design		
Cross-sectional	76	(51.7)
Retrospective cohort	23	(15.6)
Prospective cohort	42	(28.6)
RCT	6	(4.1)
BR-specific PROs		
BREAST-Q	142	(96.6)
EOQTC QLQ -BRR26	3	(2.0)
BRECON-31	2	(1.4)
Baseline survey		
Yes	51	(34.7)
No	96	(65.3)
Time points of postoperative survey		
Yes, multiple points	29	(19.7)
Yes, one point	36	(24.5)
Defined with a wide range	4	(2.7)
Only the lower limit is defined	24	(16.3)
Not defined	54	(36.7)

*PRO* patient-reported outcomes, *BR* breast reconstruction, *RCT* randomized controlled trials, *EOQTC QLQ -BRR26* the European organization for research and treatment of cancer quality of life questionnaire BRR26, *BRECON-31* breast reconstruction satisfaction questionnaire

The BREAST-Q was utilized in most of the studies. Preoperative baseline surveys were conducted in 51 (34.7%) studies. The timing of the postoperative survey was defined in 65 (44.2%) of the studies, whereas others were not clearly defined (36.7%), had only a lower limit such as “six months or more” (16.3%), or were broad to include years (2.7%).

### Study themes

The classification of the main themes of the studies is presented in Table 3. Eighty-one (55.1%) studies were classified into BR surgery-related themes. Of which these studies, 20 (13.6%) compared autologous-tissue BR (ATBR) and implant-based BR (IBBR) [15–34]. Eight (5.4%) studies focused on ATBR-specific themes, which included three studies that compared ATBR types [35–37] and five studies that focused on abdominal-based flap techniques and various topics: laterality of pedicle [38], utilization of indocyanine green angiography [39], studies on the recipient vessels [40], nerve coaptation to the sensory nerve [41], and vascularized lymph node transfer performed simultaneously [42]. IBBR-specific themes were the most common topic of 34 (23.1%) studies, which included 10 studies that compared the direct-to-implant (DTI) procedure with staged procedure [43–52], 9 that compared subpectoral DTI with and without mesh support [53–61], 7 that compared subpectoral and pre-pectoral implant insertion [62–68], 4 that compared mesh types [69–72], 2 that compared implant types [73, 74], and 2 that were related to animation deformity [75, 76]. This reflects the transition in technique from conventional sub-pectoral, two-staged procedure to pre-pectoral, direct-to-implant procedure because of the widespread use of biological matrix and synthetic mesh. Moreover, 10 studies (6.8%) focused on mastectomy, of which six compared nipple-sparing mastectomy (NSM) with non-NSM [77–82], two analyzed NSM incision [83, 84], and two evaluated two new techniques: robotic mastectomy and IBBR [85] and endoscopic-assisted NSM and latissimus dorsi flap (LD) reconstruction [86]. In addition, 3 (2.0%) studies analyzed postoperative factors, of which 2 were about complications after BR, and one was about time since surgery. Furthermore, 6 (4.1%) studies examined surgical procedures other than primary breast reconstructive surgery, of which 3 analyzed re-reconstruction after failed IBBR [87–89], 2 evaluated fat grafting [90, 91], and 1 focused on contralateral symmetry procedure [92].

**Table 3:** Classification by the main study theme

Category		Subcategory	No. of articles (%)
**BR Surgery-related themes**			**81 (55.1)**
Comparison between ATBR and IBBR			20 (13.6)
ATBR-specific theme			8(5.4)
		Comparison between the DIEP, SGAP, and LAP flap	1
		Comparison within abdominal flaps	2
		Bipedicle or unipedicle for unilateral BR	1
		ICG angiography for the DIEP flap	1
		Recipient vessels of the free abdominal flap	1
		Nerve coaptation to the sensory nerve	1
		Vascularized lymph node transfer with the DIEP flap	1
IBBR-specific theme			34 (23.1)
		Direct vs staged	10
		Subpectoral DTI with or without mesh support	9
		Subpectoral vs pre-pectoral IBBR	7
		Types of mesh	4
		Types of implants	2
		Animation deformity	2
Mastectomy			10 (6.8)
		NSM and non-NSM	6
		Incision of NSM	2
		Endoscopic and robotic approach	2
Postoperative factors			3 (2.0)
		Complications after BR	2
		Time since surgery	1
Other surgery			6 (4.1)
		Re-reconstruction after failed IBBR	3
		Fat grafting	2
		Contralateral symmetry procedure	1
**Clinical themes**			39 (26.5)
RT			20 (13.6)
		RT on ATBR and IBBR	3
		RT and ATBR	7
		RT and IBBR	10
Chemotherapy			1 (0.7)
IBR vs DBR			3 (2.0)
Laterality			5 (3.4)
HBOC and PM			8 (5.4)
		IBBR and ATBR for HBOC	1
		Contralateral PM with unilateral therapeutic MT	4
		With or without cancer in bilateral PM	3
Healthcare service			2 (1.4)
		Treating hospital	1
		Sex of the plastic surgeon	1
**Patient-related themes**			21 (14.3)
Baseline characteristics			17 (11.6)
		Race	2
		Age	3
		Obesity	6
		Overall health status	1
		Socioeconomic status	1
		Previous augmentation	1
		Pre-existing psychiatric problems	3
Postoperative factors			4 (2.7)
		Psychosocial well-being	1
		Financial toxicity	1
		Postoperative opioid consumption	1
		Disparity between patient and observer perception	1
**Exploring predictors of satisfaction and QOL**			**6 (4.1)**

*ATBR* autologous-tissue breast reconstruction, *BR* breast reconstruction, *DIEP* deep inferior epigastric artery perforator, *SGAP* superior gluteal artery perforator, *LAP* lumbar artery perforator, *ICG* indocyanine green, *DTI* direct-to-implant, *DBR* delayed breast reconstruction, *HBOC* hereditary breast and ovarian cancer syndrome, *IBBR* implant-based breast reconstruction, *IBR* immediate breast reconstruction, *PM* prophylactic mastectomy, *MT* mastectomy, *RT* radiation therapy, *QOL* quality of life

Thirty-nine (26.5%) studies were classified into the clinical theme. Of these studies, 20 (13.6%) assessed the effect of radiotherapy (RT) on ATBR [93–99], IBBR [100–109], or both [110–112]. Seven of these studies discussed the timing of BR and RT [95–99, 104, 108]. Moreover, 1 (0.7%) study examined the influence of chemotherapy [113], 3 (2.0%) compared immediate and delayed reconstruction [114–116], and 5 (3.4%) compared unilateral and bilateral reconstructions [117–121]. Eight studies with clinical themes (5.4%) focused on HBOC and PM, in which the issues to consider were complex, namely whether to perform contralateral PM in women with unilateral breast cancer [122–124] and compare them with women with bilateral breast cancer [125], comparison between BPM cases with and without previous cancer [126–128], and comparison between IBBR and ATBR for women with BRCA mutation [129]. Furthermore, 2 (1.4%) studies examined healthcare services such as the treating hospital [130] and the sex of the plastic surgeon [131].

Moreover, 21 (14.3%) studies had patient-related themes, of which 17 (11.6%) were on baseline characteristics. Obesity was the most frequently studied factor [132–137], followed by age [138–140] and pre-existing psychiatric problems [141–143]. Other baseline characteristics included race [144, 145], overall health status [146], socioeconomic status [147], and history of previous augmentation surgery [148]. Four studies (2.7%) examined the effect of postoperative patient factors such as postoperative opioid consumption [149], financial burden of BR [150], disparity between patient and observer perceptions of outcomes [151], and psychosocial well-being [152].

Six studies (4.1%) explored predictors of satisfaction and QOL after BR [153–158]. The factors extracted from these studies were nicotine dependence [155], antibody treatment [155], lymphedema [155], breast sensitivity [154], pain [154], scar thickness [154], time since surgery [153, 154], preoperative and postoperative psychosocial well-being [154, 156], preoperative sexual wellbeing [156], preoperative physical well-being of abdomen [157], reconstructive procedure [153], and race [157].

**Fig. 3:**
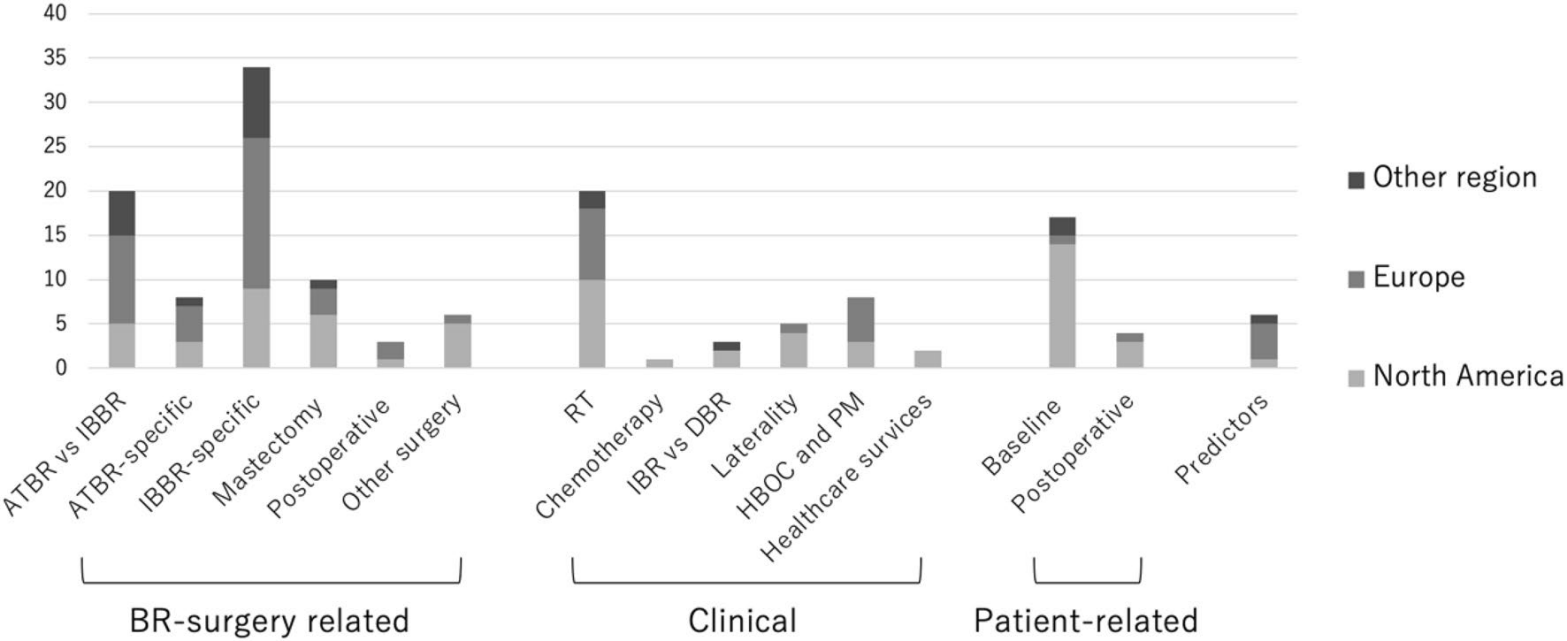
Distribution of study themes by region. *ATBR* autologous-tissue breast reconstruction, *BR* breast reconstruction, *DBR* delayed breast reconstruction, *HBOC* hereditary breast and ovarian cancer syndrome, *IBBR* implant-based breast reconstruction, *IBR* immediate breast reconstruction, *PM* prophylactic mastectomy, *RT* radiation therapy

Figure 3 shows the distribution of the study themes by world regions. Studies on reconstructive techniques and treatment strategies have been conducted in all regions; however, studies on patient factors such as obesity, race, and age have been conducted mainly in North America.

## Discussion

BR-specific PROMs developed in North America and Europe have been translated and disseminated in many countries, and the BREAST-Q is now the most used worldwide, contributing to the increase in the number and diversification of studies in satisfaction and QOL after BR.

As regards recent trends in research themes, in addition to the basic theme of autologous tissues in comparison with implants, optimization of the outcomes of each surgery by stratifying treatment factors and patient factors has been investigated. Studies have also discussed ATBR techniques based on abdominal flap BR to further improve the QOL of patients; however, the introduction of new surgical materials such as acellular dermal matrices (ADM) has led to changes in surgical techniques in IBBR, and many studies have evaluated new techniques from the perspective of patient satisfaction and QOL.

Another major focus of the world is radiation therapy. Since BR is a part of breast cancer treatment, the timing of treatment and reconstruction is a major clinical issue. As patient factors, in addition to demographic factors such as race and age, obesity, preoperative psychiatric disorders, and postoperative psychological aspects are being considered, and these studies have been conducted mainly in North America. These patient factors should be considered potential confounders in future clinical studies. In clinical practice, patient education on these factors and patient support from the preoperative to the postoperative period were suggested to improve postoperative satisfaction and QOL.

Studies using BR-specific measures were expected to accumulate and be integrated into future meta-analyses with a higher level of evidence. However, potential barriers are the quality of each study [4, 5, 159] and the heterogeneity of cultural regions [5].

Regarding the methodology of the PRO surveys analyzed in this review, only six RCTs have been conducted in five years [28, 44, 57, 58, 71, 85]. In BR, where the patient makes the decision, RCTs are difficult to conduct ethically, especially in determining surgical techniques. A strategy to compensate for the limitations of observational studies in these areas is to employ propensity-matched analysis to adjust for confounding [5], which was performed in three cross-sectional studies [24, 26, 123] and two retrospective cohort studies [30, 68]. There were 42 prospective cohort studies, of which 22 were reported in the Mastectomy Reconstruction Outcomes Consortium (MROC) study [15, 19]. MROC was conducted in 11 institutions in the United States and Canada, and PRO was evaluated longitudinally from before surgery to 2 years after surgery, and data were accumulated for various analyses including the effect of RT [93, 96, 104, 105, 111], age [138], race [144], and other factors. Such multicenter studies are a valuable reference for future clinical research. Gallo et al. stated that appropriate BREAST-Q administration, reporting of appropriate time horizon, and sample size calculations were important to ensure sufficient data quality [159]. In the present study, the time points of PRO surveys were clearly prespecified in 44.1% of the studies analyzed. Despite conflicting reports that satisfaction improves with time since surgery [115, 160] and conversely declines [23, 31, 153], the short-term and long-term results likely vary because women’s breast shape changes considerably with age, and implant-reconstructed breasts are deformed by capsular contracture. Therefore, the appropriate timing of evaluation should be determined in advance according to the purpose of the study.

Cultural backgrounds, women’s body shapes, and values differ among countries, and the response patterns and average values differ even with the same scale. Thus, the extent to which the findings of other county’s studies are applicable to Japanese populations is uncertain. They are more likely to be skinny than their Western counterparts, less likely to have large ptotic breasts, and have limited donor-site volume in the abdomen and thighs. Based on body shape, Asian studies may be more helpful for Japanese than for Westerners. For example, Cheng et al. analyzed 415 patients who underwent BR with abdominal free-flap BR in Taiwan, 76.8% were of normal weight (body mass index [BMI]; 18.5 < BMI < 24.9 kg/m^2^) and 23.2% were overweight (25 < BMI < 29.9 kg/m^2^) [137], whereas Srinivasa et al. reported that 24.3% of the 634 patients who underwent ATBR and enrolled in the MROC study were classified as normal weight, 34.5% as overweight, and 41.2% as class I or higher (29.9 kg/m^2^ < BMI) obese [136]. Differences between countries can also be seen in healthcare resources. Specifically, biomaterials such as ADMs cannot be used under Japanese health insurance; however, many of the IBBR-related studies included in this study involved cases in which ADM was used. This suggests that while the results of studies conducted in other countries are very informative, country-specific surveys and data accumulation are needed. Studies have also reported low response rates and low average values for the sexual well-being of Japanese women based on BREAST-Q [158, 161], and Japanese may have even lower scores than other Asian women [161]. A trend was found toward generating normative data for the interpretation of BREAST-Q [162–164]. Crittenden et al. reported that the Australian normative values were significantly lower than the US normative values on four of the five subscales [162], suggesting cultural and racial differences. Future work will require the creation of normative data in Japan to better understand the effect of BR.

This scoping review was conducted to map what studies on satisfaction and QOL after mastectomy and BR have been conducted, which demonstrated the increasing contribution of BR-specific PROMs worldwide and implied the need for further research in their respective culture using in appropriate methodology.

This study has several limitations. It dealt only with studies that measured postoperative satisfaction or QOL after mastectomy and BR. Therefore, important topics that may have influenced QOL after reconstructive surgery such as oncoplastic surgery [165], decision aids [166], and expectation management [167, 168] were not included in the analysis. Similarly, studies that utilized other valuable PROMs to investigate perioperative pain management, decision regrets, overall health status, etc., were excluded. The authors also recognize the need to consider sample size calculations [159] and minimally important difference [169] estimation in planning future studies using PROs.

In conclusion, recent BR-specific PROMs have led to a worldwide development of research on factors that affect satisfaction and QOL after BR, including a wide range of surgical, clinical, and patient factors. PROs after BR may be influenced by local cultural and social features; thus, accumulating data in each region is necessary.
